# *KRAS* and *NRAS* mutations in Nordic population-based and real-world metastatic colorectal cancer cohorts

**DOI:** 10.1038/s44276-025-00188-5

**Published:** 2025-10-21

**Authors:** Emerik Osterlund, Ari Ristimäki, Luís Nunes, Soili Kytölä, Sonja Aho, Eetu Heervä, Aki Uutela, Kaisa Lehtomäki, Hanna Stedt, Päivi Halonen, Joel Kontiainen, Timo Muhonen, Raija Kallio, Jari Sundström, Annika Ålgars, Raija Ristamäki, Lasse Nieminen, Halfdan Sorbye, Per Pfeiffer, Tapio Salminen, Arno Nordin, Annamarja Lamminmäki, Markus J. Mäkinen, Tobias Sjöblom, Helena Isoniemi, Bengt Glimelius, Pia Osterlund

**Affiliations:** 1 Department of Immunology, Genetics and Pathology, Uppsala University, Uppsala, Sweden; 2 Department of Oncology, University of Turku and Turku University Hospital, Turku, Finland; 3 Department of Pathology, HUSLAB, HUS Diagnostic Center, Helsinki University Hospital, Helsinki, Finland; 4 Applied Tumor Genomics Research Program, Research Programs Unit, Faculty of Medicine, University of Helsinki, Helsinki, Finland; 5 Department of Molecular Oncology, Institute for Cancer Research, Oslo University Hospital, Oslo, Norway; 6 Department of Genetics, HUSLAB, HUS Diagnostic Center, Helsinki UniversiIty Hospital, Helsinki, Finland; 7 Department of Genetics, University of Helsinki, Helsinki, Finland; 8 Department of Oncology, Tampere University Hospital, Tampere, Finland; 9 Faculty of Medicine and Health Technology, Tampere University, Tampere, Finland; 10 Department of Transplantation and Liver Surgery, Helsinki University Hospital, Helsinki, Finland; 11 Department of Surgery, University of Helsinki, Helsinki, Finland; 12 Department of Oncology, Kuopio University Hospital, Kuopio, Finland; 13 Faculty of Health Sciences, University of Eastern Finland, Kuopio, Finland; 14 Department of Oncology, Helsinki University Hospital, Helsinki, Finland; 15 Department of Oncology, University of Helsinki, Helsinki, Finland; 16 Department of Oncology, South Karelia Central Hospital, Lappeenranta, Finland; 17 Department of Oncology, Oulu University Hospital, Oulu, Finland; 18 Department of Oncology, University of Oulu, Oulu, Finland; 19 Department of Pathology, Turku University Hospital, Turku, Finland; 20 Institute of Biomedicine, University of Turku, Turku, Finland; 21 Department of Pathology, Fimlab laboratories, Tampere, Finland; 22 Department of Pathology, Tampere University, Tampere, Finland; 23 Cancer Clinic, Haukeland University Hospital, Bergen, Norway; 24 Department of Clinical Science, University of Bergen, Bergen, Norway; 25 Department of Oncology, Odense University Hospital, Odense, Denmark; 26 Department of Clinical Research, University of Southern Denmark, Odense, Denmark; 27 Department of Pathology, Oulu University Hospital, Oulu, Finland; 28 Department of Pathology, University of Oulu, Oulu, Finland; 29 Department of Gastrointestinal Oncology, Karolinska Universitetssjukhuset, Stockholm, Sweden; 30 Department of Oncology/Pathology, Karolinska Institutet, Stockholm, Sweden

## Abstract

**Background:**

*KRAS* and *NRAS* mutations (mt) are drivers in metastatic colorectal cancer (mCRC). We studied frequencies, characteristics, treatments, and outcomes of different *KRAS*mt and *NRAS*mt in population-based and real-world settings.

**Methods:**

Three Nordic cohorts were combined and molecularly characterised for *KRAS*, *NRAS*, and *BRAF*-V600E hotspot mutations.

**Results:**

Of 2649 mCRC patients, 2118 were molecularly classified. *KRAS*mt were seen in 49%, *NRAS*mt in 4%, RAS&*BRAF*wt in 33%, and *BRAF*-V600Emt in 14%. No differences in clinical characteristics were observed between *KRAS*mt and *NRAS*mt. Median overall survival (OS) was longest among RAS&*BRAF*wt, intermediate among *KRAS*mt and *NRAS*mt, and shortest among *BRAF*-V600Emt (28.3 vs 21.4 vs 26.3 vs 9.2 months, respectively). Among the eight most common *KRAS*mt, the only clinical difference was that *KRAS*-G12S had more distant lymph node metastases (38% vs 18–27%, *p* = 0.041). *KRAS*-G12S had shorter OS than *KRAS*-G12V, *KRAS*-G12C, *KRAS*-G12A, and *KRAS*-G13D. The differences were smaller in treatment groups but withstood in multivariable models. The three most common *NRAS*mt did not differ clinically.

**Conclusion:**

*KRAS*mt and *NRAS*mt are seen in 49% and 4% of mCRC, respectively. No clinically relevant differences were observed between different RASmt. *KRAS*mt is a common subgroup for which the outcome hopefully can be improved with newly developed drugs.

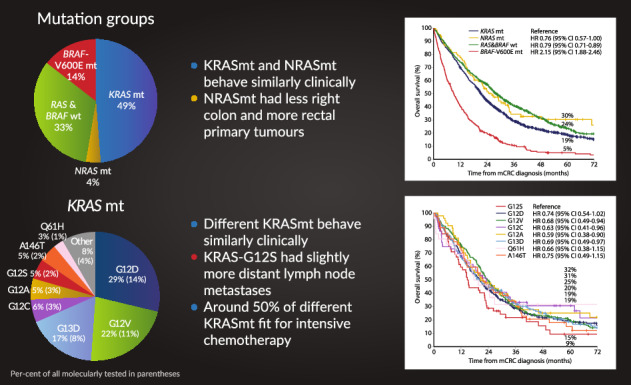

## Introduction

Colorectal cancer (CRC) is the third most common cancer and the second most common reason for cancer death [1]. Activating missense mutations (mt) in RAS genes are common drivers of cancer [2]. In CRC, *KRAS*mt and *NRAS*mt make the tumours resistant to epidermal growth factor receptor (EGFR)-inhibitors [3]. Treatment of patients with *KRAS*mt and *NRAS*mt tumours has long been restricted to conventional cytotoxic drugs, either alone or in combination with vascular endothelial growth factor inhibitors, such as bevacizumab.

*KRAS*mt are found in 32–50% of metastatic colorectal cancer (mCRC) patients (Table S1) [4–15] and in 36%–48% in mixed CRC cohorts (Table S2) [16–20]. The most common *KRAS*mt are *KRAS*-G12D (seen in 11–15% of all and 28%–40% of *KRAS*mt), followed by *KRAS*-G12V (7–10% and 20–30%, respectively) and *KRAS*-G13D (6–9% and 5–22%, respectively) [4–12, 21].

The first clinically proven effective drugs against RAS mutations were *KRAS-G12C* inhibitors. These have efficacy in mCRC when combined with EGFR inhibitors [22, 23]. Lately, inhibitors against *KRAS*-G12D have shown promising results [24]. Inhibitors targeting other specific RASmt or entire RAS genes have been developed, but their tolerability and efficacy need to be studied further [25]. Of all RASmt, *KRAS*-G12C has been studied in greater detail due to the aforementioned inhibitors [8, 11, 17, 26].

Earlier studies comparing different *KRAS*mt have shown conflicting results, with either no differences at all [5] or minor differences in clinical characteristics that are inconsistent between studies [8, 9]. This is also the case for overall survival (OS) and progression-free survival (PFS), with some studies reporting no differences between different *KRAS*mt [8], whereas others claim minor differences that again are inconsistent [4, 5, 9].

*NRAS*mt are seen in 3–6% of mCRC patients [4, 5, 12, 15, 27, 28]. In comparison with *KRAS*mt, some studies show minor differences in clinical characteristics [15], whereas others do not [27]. OS data is also conflicting, with some studies showing no difference between *NRAS*mt and *KRAS*mt [4], whereas others show an inferior OS for *NRAS*mt compared with *KRAS*mt [15, 27, 28].

Previous studies have been restricted to patients included in clinical trials, hospital-based series, and large databases, where the patients are selected. Many studies also have too few patients or lack clinical data, making it hard to draw clinically relevant conclusions. It has been shown that the frequency of certain molecular alterations differs in the background population compared with clinical trials and hospital-based series [29–32]. Earlier studies on *KRAS*-G12C in selected materials [5, 26] claimed differences that could not be verified in the background population [33]. Therefore, the representativity of what is presently known can be discussed. Knowledge about the prevalence of different *KRAS*mt and *NRAS*mt and their effects on patient characteristics, treatments given, and outcomes is sparse, especially in unselected populations.

Our aim was to study the prevalence, patient characteristics, treatments, fitness for intense therapy, and outcome for different *KRAS*mt and *NRAS*mt and how they differ in relation to RAS&*BRAF* wildtype (wt) and *BRAF*-V600Emt in population-based and real-world settings, resembling real life.

## Materials and Methods

### Cohorts and treatments

Patients with mCRC from the Finnish prospective real-world RAXO-study, a population-based cohort from the Uppsala region, Sweden, and the population-based Scandinavian prospective registration of mCRC (the PRCRC-study) were combined.

The RAXO-study included 1086 mCRC patients, 2012–2018, who were eligible for first-line chemotherapy [34]. In the cohort from the Uppsala region, all mCRC patients were prospectively identified as part of a biobank initiative, the Uppsala-Umeå Comprehensive Cancer Consortium (U-CAN), since April 2010 [35]. The remaining patients were identified retrospectively using a hospital-based registry and the Swedish ColoRectal Cancer Registry (SCRCR), resulting in a final cohort of 765 mCRC patients with a diagnosis 2010–2022 [32]. After validation against medical records, this cohort can be considered 100% complete [36]. In the PRCRC-study all 798 patients with a mCRC diagnosis from three regions around university hospitals in Norway, Denmark, and Sweden were included 2003–2006 [29].

Patients were treated according to routine clinical practice during the inclusion periods. Details in the PCRCR study have been described [29]. Treatments in the two more recent cohorts adhered to European Society for Medical Oncology (ESMO)-guidelines [37, 38].

Fitness for intensive therapy was classified according to Eastern Cooperative Oncology Group performance status (ECOG PS) 0–1 and age <75 years old, based on ESMO-guidelines [3, 38]. For this analysis only patients from the PRCRC-study and the Uppsala region cohort were included, as they were the only entirely population-based cohorts.

The STROBE statement was adhered to when conducting the study [39].

### Ethical considerations

The study was conducted according to the guidelines of the Declaration of Helsinki and approved by the Ethics Committees of Haukeland University Hospital, Helsinki University Hospital (242/13/03/02/2011 and HUS/1288/2016), Odense University Hospital, and Uppsala University (2009-408, and 2018/490).

### Informed consent statement

Written informed consent was obtained from all patients in the RAXO-study. In the Uppsala region cohort, written informed consent was obtained from all patients included in the U-CAN initiative, whereas SCRCR works through an opt-out principle. In the PRCRC-study, written informed consent was obtained from all prospectively included patients, whereas the cancer registries where the remaining patients were included from work by an opt-out principle.

### Molecular analyses

Testing for RAS and *BRAF* mutations was done in the clinical routine in the RAXO-study and for most patients in the Uppsala region cohort. Pyrosequencing or reverse transcriptase polymerase chain reaction were mostly used before 2014. From then on next next-generation sequencing (NGS) was mostly used. In the RAXO-study, some centres also used two-step Idylla testing. The composition of the NGS panels has varied over the years, but all have included analyses of *KRAS* and *NRAS* exons 2–4 (codons 12, 13, 59, 61, 117, and 146) and *BRAF*-V600E mutations according to ESMO-guidelines [37, 38]. The molecular analyses in the Uppsala region cohort were completed with whole-genome sequencing (WGS) if fresh frozen material was available [40], otherwise an NGS was done [32]. In the PRCRC-study cohort, the analyses were performed using a custom Ampliseq hotspot panel for most [31], or with pyrosequencing for *KRAS* and *BRAF*-V600E [30].

Patients who could not be accurately classified according to *KRAS*, *NRAS*, and *BRAF*-V600E were excluded from demographics and survival analyses. To be adequately classified, the presence of *KRAS*, *NRAS*, or *BRAF*-V600E mutations (assuming mutual exclusivity), was sufficient. To be classified as RAS&*BRAF* wildtype [wt], testing of *KRAS* exons 2–4, *NRAS* exons 2–4, and *BRAF*-V600E was generally required; however, 37 patients were tested only for *KRAS* and *BRAF*-V600E mutations and included in the RAS&*BRAF*wt group. No molecular tests were performed in 389 (15%) patients, and 131 (5%) could not be adequately characterised due to missing *BRAF*-V600E testing. Another 11 patients could not be adequately grouped due to *KRAS*mt and *NRAS*mt in 6, RASmt and *BRAF*-V600E/Rmt in 3, and unknown *KRAS*mt in 2.

Mismatch repair (MMR)-status testing in the PRCRC study has been described previously [31]. In the RAXO-study MMR testing was performed with immunohistochemistry in routine healthcare. In the Uppsala region cohort, MMR testing was done either using immunohistochemistry, with WGS [40], or with the TrueMark^TM^ MSI Assay kit (Thermo-Fisher Scientific, MA, USA) [32].

### Statistics

Categorical variables were compared using Chi-square. The Kruskal-Wallis test was used for comparing non-normally distributed continuous variables. OS was estimated using the Kaplan-Meier method and was defined as the time from diagnosis of mCRC to time of death or censored if alive at last follow-up (October 7, 2020, in RAXO, March 16, 2023, in the Uppsala region cohort, and August 17, 2008, in PRCRC). Median follow-up time was estimated using the reverse Kaplan-Meier method. PFS was estimated for patients receiving systemic therapy only using the Kaplan-Meier method and was defined as the time from treatment initiation to progression or censored if no progression at last follow-up. OS and PFS comparisons were done using Cox regression. The proportional hazard assumption was tested using Schoenfeld residuals; no clear violations were seen. A multivariable Cox regression model adjusting for other clinically relevant and statistically significant variables was constructed. Two-sided p-values < 0.05 and hazard ratios not crossing 1 were considered statistically significant. All analyses were performed using SPSS Statistics version 29 (IBM Corporation, Armonk, NY, USA).

## Results

### Patient characteristics across the study cohorts

Characteristics of all 2649 patients included in the RAXO-study, the Uppsala region cohort, and the PRCRC-study are presented in Table S3. The patients in the population-based Uppsala region and PRCRC-study cohorts were slightly older, more often female, had more right colon primary tumours, and worse ECOG PS compared with treatable patients in the RAXO-study. Median OS (mOS) was shortest in the PRCRC-study cohort, intermediate in the Uppsala region cohort, and longest in the RAXO-study. Stratified by treatment groups (cytotoxics only, cytotoxics combined with bevacizumab/EGFR-inhibitors, metastasectomy and/or local ablative therapy [LAT], and best supportive care [BSC] only), the OS differences were smaller or absent.

In total, 2118 patients were adequately tested for *KRAS*, *NRAS*, and *BRAF*-V600E mutations in the cohorts; their characteristics are shown in Table S4. The proportion bearing mutations in *KRAS* and *NRAS* was similar between cohorts. The *BRAF*-V600Emt frequency was higher in the Uppsala region cohort (17%) and the PRCRC-study (19%) compared with the RAXO-study (10%). Median follow-up was 61.8 months. OS differences were seen between the cohorts, but these were smaller when comparing the above-mentioned treatment groups separately. Therefore, we concluded that it was adequate to combine the materials to increase the number of patients with different *KRAS*mt, enabling firmer conclusions.

**Fig. 1:**
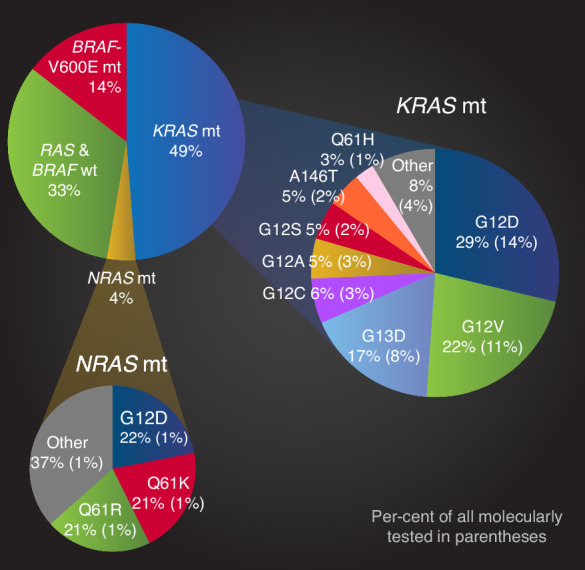
Distribution of mutation groups and the most common *KRAS* and *NRAS* mutations and their respective prevalence in each mutation group and among all molecularly tested.

The *KRAS*mt frequency was 49% and the *NRAS*mt frequency was 4% among all molecularly tested (Table 1, Fig. 1). The frequencies were 53% and 4% in the RAXO-study, 47% and 4% in the Uppsala region cohort, and 43% and 4% in the PRCRC-study, respectively (Table S4).

**Table 1:** Clinical characteristics according to mutation status.

		Total		*KRAS*mt		*NRAS*mt		RAS&*BRAF*wt		*BRAF*-V600Emt		*p*-value
		2118	100%	1033	49%	82	4%	696	33%	307	14%	
Median age (range)		69 (21–99)		69 (21–99)		66 (30–92)		67 (22–95)		71 (33–96)		<0.001
Total		2118	100%	1033	100%	82	100%	696	100%	307	100%	—
Age groups	≤70 years	1168	55%	548	53%	51	62%	423	61%	146	48%	<0.001
	>70 years	950	45%	485	47%	31	38%	273	39%	161	52%	
Sex	Male	1180	56%	584	57%	46	56%	430	62%	120	39%	<0.001
	Female	938	44%	449	43%	36	44%	266	38%	187	61%	
Primary tumour location	Right colon	712	34%	345	34%	10	12%	138	20%	219	72%	<0.001
	Left colon	695	33%	307	30%	30	37%	300	43%	58	19%	
	Rectum	695	33%	372	36%	42	51%	254	37%	27	9%	
	Multiple/unknown	16	—	9	—	—	—	4	—	3	—	—
Primary resection	No	548	26%	280	27%	23	28%	159	23%	86	28%	0.166
	Yes	1570	74%	753	73%	59	72%	537	77%	221	72%	
Tumour grade	Low	1407	77%	730	83%	58	83%	482	79%	137	49%	<0.001
	High	430	23%	150	17%	12	17%	126	21%	142	51%	
	Not available	281	—	153	—	12	—	88	—	28	—	—
Presentation of metastases	Synchronous	1306	62%	646	63%	53	65%	416	60%	191	62%	0.629
	Metachronous	812	38%	387	37%	29	35%	280	40%	116	38%	
Number of metastatic sites	1	1034	49%	505	49%	41	50%	347	50%	141	46%	0.912
	2	709	33%	348	34%	25	30%	225	32%	111	36%	
	3–6	375	18%	180	17%	16	20%	124	18%	55	18%	
Metastatic sites	Liver	1448	68%	716	69%	61	74%	509	73%	162	53%	<0.001
	Lung	697	33%	405	39%	28	34%	182	26%	82	27%	<0.001
	Lymph nodes	562	27%	242	23%	22	27%	183	26%	115	37%	<0.001
	Peritoneum	467	22%	200	19%	15	18%	145	21%	107	35%	<0.001
	Bone	83	4%	43	4%	2	2%	27	4%	11	4%	0.865
	Other	267	13%	118	11%	11	13%	91	13%	47	15%	0.316
ECOG PS	0	685	32%	345	33%	34	41%	233	33%	73	24%	<0.001
	1	880	42%	443	43%	35	43%	293	42%	109	36%	
	2–4	552	26%	244	24%	13	16%	170	24%	125	41%	
	Not available	1	—	1	—	—	—	—	—	—	—	—
Smoking status	No	581	52%	305	56%	24	55%	194	51%	58	39%	0.004
	Yes	533	48%	238	44%	20	45%	186	49%	89	61%	
	Not available	1004	—	490	—	38	—	316	—	160	—	—
Mismatch repair status	pMMR	1267	92%	616	97%	43	93%	455	96%	153	71%	<0.001
	dMMR	107	8%	19	3%	3	7%	21	4%	64	29%	
	Not tested	744	—	398	—	36	—	220	—	90	—	—
Type of treatment	Metastasectomy	586	28%	292	28%	29	35%	232	33%	33	11%	<0.001
	Systemic therapy only	1168	55%	569	55%	46	56%	364	52%	189	62%	
	Best supportive care	364	17%	172	17%	7	9%	100	14%	85	28%	

*dMMR* deficient mismatch repair, *ECOG PS* Eastern Cooperative Oncology Group performance status, *MMR* mismatch repair, mt mutated, *pMMR* proficient mismatch repair, *wt* wildtype.

When characterizing molecular subgroups based on eligibility for different treatments according to ESMO-guidelines [3] (*KRAS*mt, *NRAS*mt, and dMMR shown separately) among the same 2118 patients, 552 (26%) were left-sided RAS&*BRAF*wt (EGFR-inhibitors), 129 (6%) right-sided RAS&*BRAF*wt, 1014 (48%) were *KRAS*mt (*KRAS*-/RAS-inhibitors), 79 (4%) were *NRAS*mt (RAS-inhibitors), 243 (11%) were *BRAF*-V600Emt (chemotherapy + encorafenib + cetuximab), and 101 (5%) were dMMR (checkpoint inhibitors). If only considering patients with known MMR-status (*n* = 1374), dMMR was slightly more common, whereas the other groups largely remained unchanged: 374 (27%) were left-sided RAS&*BRAF*wt, 87 (6%) were right-sided RAS&*BRAF*wt, 616 (45%) were *KRAS*mt, 43 (3%) were *NRAS*mt, 153 (11%) were *BRAF*-V600Emt, and 101 (7%) were dMMR.

### Clinical characteristics, treatments, and outcomes for mutation groups

Characteristics for *KRAS*mt, *NRAS*mt, RAS&*BRAF*wt, and *BRAF*-V600Emt are shown in Table 1. No differences were observed between *KRAS*mt and *NRAS*mt, except for right colon primary tumours being less common among *NRAS*mt. Patients with *BRAF*-V600Emt were older, more often female, had more right colon primary tumours, high-grade tumours, and worse ECOG PS compared with the other mutation groups. *BRAF*-V600Emt was also more common in smokers and was dMMR more often. Liver metastases were less common, and distant lymph node and peritoneal metastases were more common among *BRAF*-V600Emt. Lung metastases, on the other hand, were more common in *KRAS*mt and *NRAS*mt compared with RAS&*BRAF*wt and *BRAF*-V600Emt.

Metastasectomies/LATs were performed most often among RAS&*BRAF*wt and *NRAS*mt, intermediate in *KRAS*mt, and least often among *BRAF*-V600Emt, whereas the opposite was true for BSC only (Table 1). Among patients treated with systemic therapy, fewer lines of therapy were given to patients with *BRAF*-V600Emt (Table S5). No major differences were seen in the cytotoxic agents used in the first or any line. Bevacizumab was used more often and EGFR-inhibitors, for obvious reasons, less often among *KRAS*mt, *NRAS*mt, and *BRAF*-V600Emt. Responses to first-line therapy were more common in *KRAS*mt, *NRAS*mt, and RAS&*BRAF*wt compared with *BRAF*-V600Emt.

The proportion considered fit for intensive therapy (ECOG PS 0–1 and <75 years old) was 48% for *KRAS*mt, 67% for *NRAS*mt, 51% for RAS&*BRAF*wt, and 36% for *BRAF*-V600Emt (Table S6). In treatment groups according to guidelines 53% were fit among left/sided RAS&*BRAF*wt, 42% among right-sided RAS&*BRAF*wt, 48% among *KRAS*mt, 66% among *NRAS*mt, 38% among *BRAF*-V600Emt, and 38% among dMMR (Table S6).

**Fig. 2:**
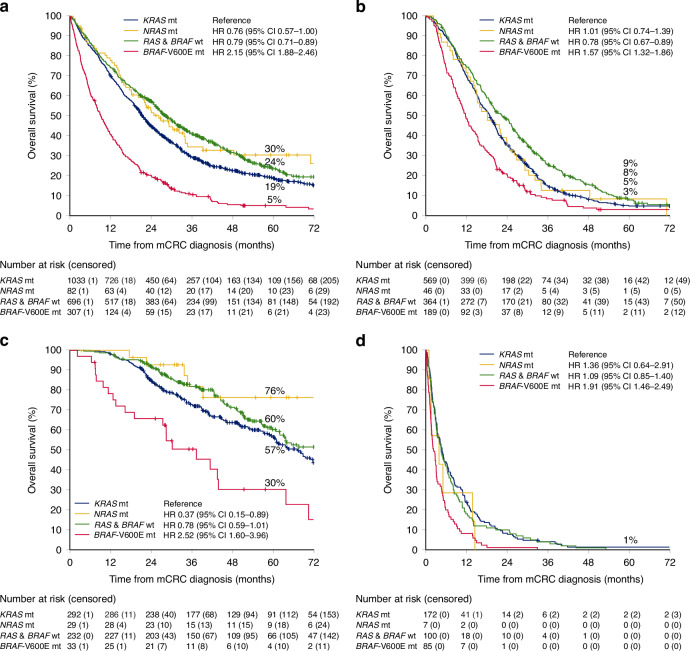
Overall survival for mutation groups. For all patients (**a**), for patients treated with systemic therapy only (**b**), for patients treated with metastasectomy and/or local ablative therapy (**c**), and for patients receiving best supportive care only (**d**).

The best OS was seen in patients with RAS&*BRAF*wt followed by *NRAS*mt and *KRAS*mt, and the shortest OS among *BRAF*-V600Emt (mOS 28.3 vs 26.3 vs 21.4 vs 9.2 months, respectively, Fig. 2A). Similar results were seen in different treatment groups (systemic therapy only, metastasectomy/LAT, BSC only), except for *NRAS*mt having more similar OS as *KRAS*mt in those treated with systemic therapy only (Fig. 2B–D). In a multivariable model adjusting for clinically relevant factors statistically significant in univariable analyses, *KRAS*mt had similar OS as *NRAS*mt, inferior compared with RAS&*BRAF*wt, but better than *BRAF*-V600Emt (Table 2A).

**Table 2:** Univariable and multivariable cox regression model for overall survival for mutation groups (A) and the most common *KRAS* mutants (B).

A								
			Univariable			Multivariable		
		*N*	HR	95% CI	*p*-value	HR	95% CI	*p*-value
Age		2117	1.03	1.02–1.03	<0.001	0.99	0.99–1.00	0.010
Primary tumour location	Right colon	712	1			1		
	Left colon	694	0.66	0.59–0.74	<0.001	0.95	0.84–1.08	0.433
	Rectum	695	0.64	0.57–0.72	<0.001	0.91	0.80–1.03	0.129
	Unknown	16	1.59	0.96–2.66	0.075	1.45	0.87–2.44	0.159
Tumour grade	Low	1407	1			1		
	High	430	1.88	1.67–2.11	<0.001	1.57	1.39–1.78	<0.001
	Not available	280	1.63	1.42–1.87	<0.001	1.33	1.15–1.54	<0.001
Number of metastatic sites	1	1033	1			1		
	2	709	1.72	1.54–1.92	<0.001	1.33	1.19–1.49	<0.001
	3–5	375	2.21	1.94–2.52	<0.001	1.64	1.43–1.87	<0.001
ECOG PS*	0	685	1			1		
	1	880	1.67	1.48–1.88	<0.001	1.42	1.26–1.61	<0.001
	2–4	552	4.40	3.86–5.01	<0.001	2.35	2.04–2.71	<0.001
Type of treatment	Systemic therapy only	1168	1			1		
	Metastasectomy	586	0.21	0.18–0.24	<0.001	0.27	0.23–0.31	<0.001
	Best supportive care	363	3.53	3.12–4.00	<0.001	3.42	2.92–4.00	<0.001
Mismatch repair status	pMMR	1266	1			1		
	dMMR	107	1.64	1.33–2.03	<0.001	0.69	0.55–0.87	0.002
	Not tested	755	0.99	0.89–1.10	0.840	0.97	0.87–1.08	0.556
Mutation groups	*KRAS*mt	1032	1			1		
	*NRAS*mt	82	0.76	0.57–1.00	0.050	0.91	0.69–1.21	0.526
	*RAS*&*BRAF*wt	696	0.79	0.71–0.89	<0.001	0.81	0.72–0.90	<0.001
	*BRAF*-V600Emt	307	2.15	1.88–2.47	<0.001	1.72	1.48–2.00	<0.001
**B**								
Age		945	1.02	1.01–1.03	<0.001	0.99	0.98–1.00	0.008
Primary tumour location	Right colon	314	1			1		
	Left colon	288	0.82	0.69–0.99	0.033	0.85	0.71–1.02	0.081
	Rectum	335	0.81	0.68–0.96	0.016	0.90	0.75–1.07	0.231
	Unknown	8	1.51	0.71–3.19	0.286	0.92	0.42–2.05	0.845
Tumour grade	Low	671	1			1		
	High	138	1.75	1.44–2.13	<0.001	1.70	1.39–2.08	<0.001
	Not available	136	1.55	1.27–1.89	<0.001	1.36	1.10–1.68	0.005
Number of metastatic sites	1	460	1			1		
	2	319	1.84	1.56–2.16	<0.001	1.28	1.09–1.52	0.004
	3–5	166	2.21	1.82–2.69	<0.001	1.56	1.27–1.91	<0.001
ECOG PS*	0	322	1			1		
	1	407	1.63	1.37–1.94	<0.001	1.44	1.20–1.71	<0.001
	2–4	216	4.40	3.62–5.36	<0.001	2.86	2.27–3.59	<0.001
Type of treatment	Systemic therapy only	519	1			1		
	Metastasectomy	268	0.21	0.17–0.26	<0.001	0.26	0.21–0.32	<0.001
	Best supportive care	158	2.90	2.41–3.49	<0.001	2.86	2.27–3.59	<0.001
*KRAS*mt	G12S	52	1			1		
	G12D	296	0.74	0.54–1.02	0.067	0.69	0.50–0.96	0.026
	G12V	231	0.68	0.49–0.94	0.018	0.63	0.46–0.88	0.007
	G12C	60	0.63	0.41–0.96	0.031	0.56	0.37–0.86	0.007
	G12A	54	0.59	0.38–0.90	0.016	0.75	0.49–1.16	0.197
	G13D	179	0.69	0.49–0.97	0.032	0.67	0.48–0.94	0.021
	Q61H	26	0.66	0.38–1.16	0.146	0.83	0.47–1.45	0.510
	A146T	47	0.75	0.49–1.15	0.191	0.85	0.55–1.32	0.468

*1 patient with missing ECOG PS was not included in the Cox regression models, *CI* confidence interval, *dMMR* deficient mismatch repair, ECOG PS European Cooperative Oncology Group performance status, *HR* hazard ratio, *mt* mutation, *N* number of patients, *pMMR* proficient mismatch repair, *wt* wildtype.

*KRAS*mt had similar PFS as *NRAS*mt and shorter PFS than RAS&*BRAF*wt and better PFS than *BRAF*-V600Emt (mOS 6.9 vs 6.9 vs 7.9 vs 4.7 months, Fig. S1A).

### Prevalence of different *KRAS* and *NRAS* mutations

The prevalences of the most common *KRAS*mt and *NRAS*mt are presented in Fig. 1. All *KRAS*mt and *NRAS*mt identified in the cohorts are presented in Table S1.

### Clinical characteristics, treatments, and outcomes for different *KRAS* mutations

Clinical characteristics for the eight most common *KRAS*mt are presented in Table 3. No clinical differences were seen between the eight most common *KRAS*mt, except for distant lymph node metastases being more prevalent among *KRAS*-G12S compared with the other *KRAS*mt (38% vs 18%–27%, *p* = 0.041).

**Table 3:** Clinical characteristics according to the eight most common *KRAS* mutations.

		Total		G12D		G12V		G12C		G12A		G12S		G13D		Q61H		A146T		p-value
		1033	100%	297	29%	231	22%	60	6%	54	5%	52	5%	179	17%	26	3%	47	5%	
Median age (range)		69 (21–99)		70 (21–91)		68 (40–92)		69 (35–90)		68 (23–91)		71 (38–91)		70 (29–99)		65 (46–85)		67 (35–97)		0.833
Total		1033	100%	297	100%	231	100%	60	100%	54	100%	52	100%	179	100%	26	100%	47	100%	—
Age groups	≤70 years	548	53%	145	49%	133	58%	33	55%	33	61%	24	46%	90	50%	16	62%	30	64%	0.188
	>70 years	485	47%	152	51%	98	42%	27	45%	21	39%	28	54%	89	50%	10	38%	17	36%	
Sex	Male	584	57%	169	57%	130	56%	36	60%	31	57%	29	56%	104	58%	12	46%	26	55%	0.976
	Female	449	43%	128	43%	101	44%	24	40%	23	43%	23	44%	75	42%	14	54%	21	45%	
Primary tumour location	Right colon	345	34%	109	37%	71	31%	15	25%	16	30%	11	21%	69	39%	5	19%	18	39%	0.198
	Left colon	307	30%	87	29%	65	29%	22	37%	22	41%	21	40%	52	29%	10	38%	10	22%	
	Rectum	372	36%	99	34%	90	40%	23	38%	16	30%	20	38%	58	32%	11	42%	18	39%	
	Multiple/unknown	9	—	2	—	5	—	—	—	—	—	—	—	—	—	—	—	1	—	—
Primary resection	No	280	27%	83	28%	63	27%	16	27%	11	20%	16	31%	44	25%	7	27%	14	30%	0.932
	Yes	753	73%	214	72%	168	73%	44	73%	43	80%	36	69%	135	75%	19	73%	33	70%	
Tumour grade	Low	730	83%	209	82%	165	83%	41	84%	40	91%	39	83%	128	82%	19	83%	30	81%	0.945
	High	150	17%	45	18%	34	17%	8	16%	4	9%	8	17%	28	18%	4	17%	7	19%	
	Not available	153	—	43	—	32	—	11	—	10	—	5	—	23	—	3	—	10	—	—
Presentation of metastases	Synchronous	646	63%	179	60%	150	65%	38	63%	38	70%	32	62%	110	61%	16	62%	29	62%	0.904
	Metachronous	387	37%	118	40%	81	35%	22	37%	16	30%	20	38%	69	39%	10	38%	18	38%	
Number of metastastic sites	1	505	49%	134	45%	115	50%	33	55%	30	56%	25	48%	82	46%	17	65%	25	53%	0.340
	2	348	34%	109	37%	78	34%	19	32%	19	35%	14	27%	61	34%	3	12%	16	34%	
	3–6	180	17%	54	18%	38	16%	8	13%	5	9%	13	25%	36	20%	6	23%	6	13%	
Metastatic sites	Liver	716	69%	204	69%	164	71%	43	72%	36	67%	39	75%	120	67%	15	58%	36	77%	0.691
	Lung	405	39%	115	39%	89	39%	23	38%	22	41%	18	35%	77	43%	10	38%	19	40%	0.974
	Lymph nodes	242	23%	73	25%	41	18%	13	22%	11	20%	20	38%	49	27%	8	31%	8	17%	0.041
	Peritoneum	200	19%	66	22%	48	21%	7	12%	8	15%	8	15%	36	20%	3	12%	7	15%	0.423
	Bone	43	4%	13	4%	12	5%	2	3%	1	2%	2	4%	8	4%	1	4%	0	0%	0.823
	Other	118	11%	39	13%	26	11%	6	10%	4	7%	2	4%	19	11%	6	23%	4	9%	0.260
ECOG PS	0	345	33%	102	34%	71	31%	16	27%	22	41%	19	37%	62	35%	9	35%	21	45%	0.172
	1	443	43%	126	43%	114	49%	22	37%	20	37%	19	37%	72	40%	13	50%	21	45%	
	2–4	244	24%	68	23%	46	20%	22	37%	12	22%	14	27%	45	25%	4	15%	5	11%	
	Not available	1	—	1	—	—	—	—	—	—	—	—	—	—	—	—	—	—	—	—
Smoking status	No	305	56%	81	53%	69	60%	26	72%	17	55%	12	46%	48	49%	7	50%	14	64%	0.279
	Yes	238	44%	73	47%	46	40%	10	28%	14	45%	14	54%	49	51%	7	50%	8	36%	
	Not available	490	—	143	—	116	—	24	—	23	—	26	—	82	—	12	—	25	—	—
Mismatch repair status	pMMR	616	97%	184	97%	142	99%	24	96%	33	97%	32	100%	111	97%	19	95%	25	89%	0.275
	dMMR	19	3%	6	3%	2	1%	1	4%	1	3%	0	0%	3	3%	1	5%	3	11%	
	Not tested	398	—	107	—	87	—	35	—	20	—	20	—	65	—	6	—	19	—	—
Type of treatment	Metastasectomy	292	28%	89	30%	63	27%	19	32%	17	31%	10	19%	46	26%	7	27%	17	36%	0.285
	Systemic therapy only	569	55%	151	51%	137	59%	28	47%	30	56%	33	63%	108	60%	12	46%	20	43%	
	Best supportive care	172	17%	57	19%	31	13%	13	22%	7	13%	9	17%	25	14%	7	27%	10	21%	

*87 patients with less common *KRAS* mutations were not presented separately and not included in the chi-square analyses, *dMMR* deficient mismatch repair, *ECOG PS* Eastern Cooperative Oncology Group performance status, *pMMR* proficient mismatch repair.

No significant differences in type of treatment (systemic therapy only, metastasectomy/LAT, BSC only) were seen between different *KRAS*mt (Table 3). *KRAS*-G12S had numerically lower metastasectomy/LAT rates compared with the other *KRAS*mt (19% vs 21–36%). The number of treatment lines was the fewest in *KRAS*-Q61H, followed by *KRAS*-G12C and *KRAS*-G12S as compared with other *KRAS*mt (≥3 lines in 5% vs 26% vs 28% vs 32–38%, *p* = 0.019), with the caveat of a few patients in the *KRAS*-Q61H subgroup (Table S7). No differences were seen for drugs used in first-line or response to first-line therapy. Bevacizumab was used less in any line among *KRAS*-G12C and *KRAS*-G12S compared with the other *KRAS*mt (44% vs 44% vs 56–84%, *p* = 0.003).

The frequencies of patients considered fit for intense therapy varied between 45–63% for all *KRAS*mt except *KRAS*-G12C, where it was 27%, with the caveat of this subgroup being small (Table S6).

**Fig. 3:**
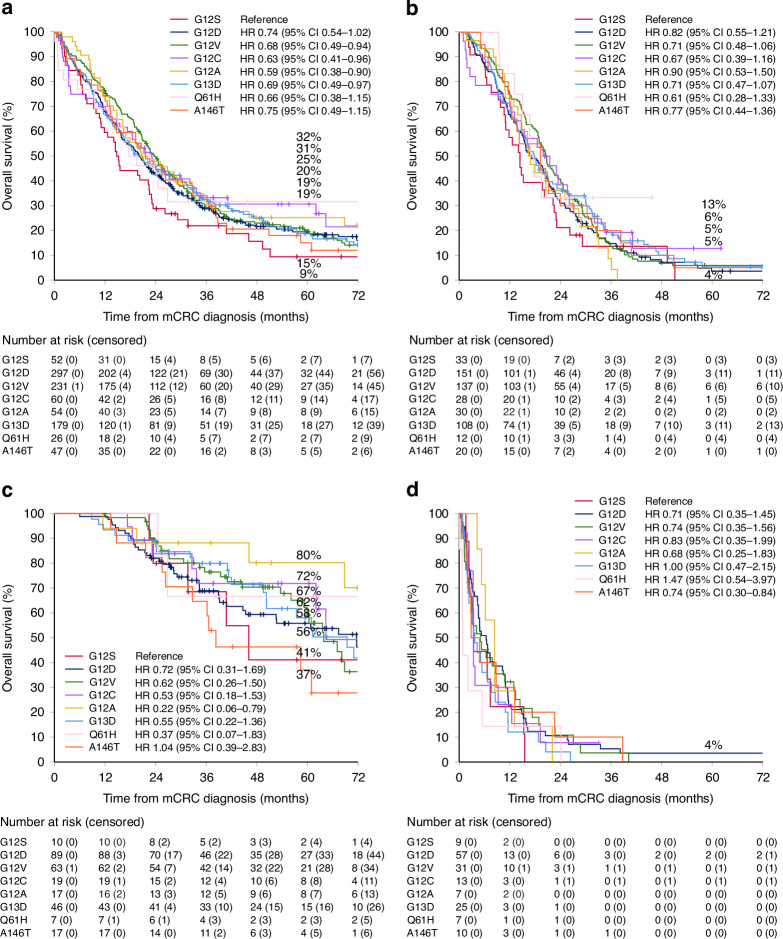
Overall survival for different *KRAS* mutations. For all patients (**a**), for patients treated with systemic therapy only (**b**), for patients treated with metastasectomy and/or local ablative therapy (**c**), and for patients receiving best supportive care only (**d**).

OS varied between 14.8–23.7 for different *KRAS*mt. *KRAS*-G12S had a statistically significantly worse OS compared with *KRAS*-G12V, *KRAS*-G12C, *KRAS*-G12A, and *KRAS*-G13D, whereas no differences were seen between any other *KRAS*mt (Fig. 3A). In treatment groups, the differences were much smaller, and the only statistically significant difference was an inferior OS for *KRAS*-G12S compared with *KRAS*-G12A in the metastasectomy/LAT group (Fig. 3B–D). In a multivariable model adjusting for clinically relevant factors statistically significant in univariable analyses similar results were seen, except for *KRAS*-G12D doing significantly better than *KRAS*-G12S and *KRAS*-G12A no longer having a significantly better OS than *KRAS*-G12S (Table 2B).

Numerically *KRAS*-G12S and *KRAS*-G12A had the shortest PFS compared with other *KRAS*mt (mPFS 6.0 vs 6.0 vs 6.4–9.2 months), however, no differences were statistically significant (Fig. S1b).

### Clinical characteristics, treatments, and outcomes for different *NRAS* mutations

Clinical characteristics for the three most common *NRAS*mt are shown in Table S8. As *NRAS*mt are rare the patient numbers are limited in all subgroups and some analyses were therefore not performed. Patients with *NRAS*-G12D were youngest, *NRAS*-Q61R intermediate, and *NRAS*-Q61K oldest. Metachronous metastases were more common among *NRAS*-Q61K compared with *NRAS*-G12D and *NRAS*-Q61R, whereas no other differences were seen.

Treatment and the proportions of different treatment groups did not differ between *NRAS*mt (Tables S8 and S9).

No differences in OS were seen between *NRAS*mt (mOS 22.0 months for *NRAS*-G12D, 20.2 months for *NRAS*-Q61K, and 26.9 months for *NRAS*-Q61R, Fig. S2). Due to the small numbers of patients, no subgroup analyses by treatment group or for PFS among systemic therapy only were done.

## Discussion

In this large population-based and real-world mCRC cohort, *KRAS*mt were seen in 49% and *NRAS*mt in 4% of molecularly tested tumours. *KRAS*mt and *NRAS*mt did not differ clinically or regarding treatments provided. However, some differences were seen when they were compared with RAS&*BRAF*wt, and especially with *BRAF*-V600Emt as could be expected. *KRAS*mt had a worse OS than RAS&*BRAF*wt, but better than *BRAF*-V600Emt. The clinical behaviour for different *KRAS*mt was similar with the only difference being *KRAS*-G12S having more distant lymph node metastases and a slightly worse OS compared with some other *KRAS*mt. Different *NRAS*mt also behaved similarly.

The *KRAS*mt frequency of 49% is higher than in most previous studies (32%–43%) mainly reporting frequencies in selected mCRC populations (Table S1) [4–7, 10–12], and similar to one other study (50%) [15]. The *KRAS* testing was more extensive for most patients in our material than in some other studies and included hotspot mutations in codons 12, 13, 59, 61, 117, and 146, as is presently recommended [3]. We have shown that certain traits with poorer prognosis, such as *BRAF*-V600Emt, dMMR, and right-sided primaries, are more common in non-selected populations than in study populations and hospital-based series [30–32]. As *KRAS*mt have at least a slightly worse prognosis than RAS&*BRAF*wt, that is one possible explanation for the higher frequency in real-life or population-based cohorts. *NRAS*mt were seen in 4%, in line with 3–6% in other cohorts [4, 5, 12, 15, 27, 28]. *BRAF*-V600Emt was seen in 14% (20% in the two population cohorts, 10% in the real-life cohort), which is more common than in selected hospital-based cohorts [3, 4, 12] and in line with population-based materials [30–32]. RAS&*BRAF*wt were seen in 33%, which is less frequent than in selected materials [4, 12] and explained by higher RASmt and *BRAF*-V600Emt frequencies.

*KRAS*mt patients were relatively similar to RAS&*BRAF*wt in this study, except for being slightly older, being female more often, and having right colon primary tumours and lung metastases more often. This is in line with Schirripa et al. [15]. Modest et al. have shown similar results for sex and lung metastases, but not for age [4]. He et al. also reported more right colon primary tumours among *KRAS*mt, but contrary to us report no difference in lung metastases, but more peritoneal metastases among *KRAS*mt [12].

Regarding clinical characteristics, *KRAS*mt and *NRAS*mt were similar except for rectal primary tumours being more common among *NRAS*mt. In other studies, the clinical characteristics have been similar as well, except that none of them saw the same results for primary location [4, 15, 27, 28]. One study showed fewer lung metastases in *NRAS*mt compared with *KRAS*mt [15]. No differences were seen between *NRAS*mt and RAS&*BRAF*wt in this study, which was also the case in other studies [4, 15, 27, 28]. Contrary to our results, one study showed more right colon primary tumours and female sex among *NRAS*mt [27], whereas another showed that *NRAS*mt were left-sided more often and more common among African Americans [28]. Given the low frequency of *NRAS*mt and the different results from the studies, the general conclusion is that *KRAS*mt and *NRAS*mt do not reveal clinically relevant differences.

Metastasectomies/LATs were done most often in RAS&*BRAF*wt and *NRAS*mt, intermediate in *KRAS*mt, and least often in *BRAF*-V600Emt, whereas the opposite was true for BSC only. Most studies have not reported the proportion receiving metastasectomies or BSC only, which is relevant as they considerably affect OS [4, 15, 27]. Contrary to our results, Cercek et al. reported fewer metastasectomies among *NRAS*mt compared with *KRAS*mt and RAS&*BRAF*wt [28].

The better OS seen among *NRAS*mt compared with *KRAS*mt in our study is probably explained by a higher proportion having metastasectomies. Most other studies have also shown the best OS in RAS&*BRAF*wt, intermediate in *KRAS*mt, and the worst in *BRAF*-V600Emt [4, 15, 27]. However, several miss *BRAF* testing [6, 11] or report no OS data [7, 10]. Regarding *NRAS*mt one study showed similar OS to *KRAS*mt and RAS&*BRAF*wt in line with our results [4], whereas three other studies showed an inferior OS for *NRAS*mt [15, 27, 28]. PFS followed the same pattern as OS, and similar results have been reported by Modest et al. [4]. Our conclusion is that *KRAS*mt and *NRAS*mt seem to have a similar prognosis, which might be slightly inferior to RAS&*BRAF*wt.

The most common *KRAS*mt in our material were *KRAS*-G12D, followed by *KRAS*-G12V and *KRAS*-G13D. The prevalences of these and of less common mutations are in line with previous studies (Table S1) [4–12]. Therefore, it seems as if *KRAS*mt overall are more common in less selected materials, but the proportions of different *KRAS*mt are similar. The proportion of different *KRAS*mt in less selected materials should also not differ, as the clinical behaviour and prognosis is similar.

Clinically, the different *KRAS*mt behaved similarly in this study, except for *KRAS*-G12S having distant lymph node metastases more often. This is in line with most previous studies, where the first showed no differences between different *KRAS*mt [5], the second showed differences only in invasions of the primary tumour and rarer metastatic sites [8], the third showed differences only in frequency of liver and lung metastases [9], and the fourth showed differences only for sex, resection of primary tumour, and frequency of liver and peritoneal metastases [21]. None, however, showed the difference we saw for distant lymph node metastases [5, 8, 9, 21]. Regarding treatments, no significant differences in treatment groups or response to first-line therapy were seen in our study; however, some groups received fewer lines of therapy or received bevacizumab less often. The *KRAS*-G12S subgroup showed a trend for being treated with metastasectomies and/or LATs less often. Contrary to our results, Ottaiano et al. saw differences in responses to first-line chemotherapy, but no differences in the number of lines [5].

As the Uppsala region cohort and the PRCRC-study were population-based, fitness for intense therapy among different *KRAS*mt could be assessed. Patients with ECOG PS 0–1 and age <75 years were considered fit. The frequencies varied between 45–63% for all except the small *KRAS*-G12C subgroup, where it was 27%. This means that roughly half of the patients with a specific mutation could be treated with intensive therapy or that about a quarter can be treated with pan-*KRAS* or -RAS inhibitors, when developed. This is especially relevant if these inhibitors are combined with chemotherapy [41].

A similar OS was seen for different *KRAS*mt, except for *KRAS*-G12S, which had slightly worse OS. Other studies have shown an inferior OS for *KRAS*-G12S and/or *KRAS*-G12C [4, 5, 9]. Two other studies saw no difference in OS between different *RAS*mt [8, 14]. PFS was similar for different *KRAS*mt in this study, which is in line with Giampieri et al. [8], whereas another study showed longer PFS for *KRAS*-G12D compared with *KRAS*-G12C but not in comparison with other *KRAS*mt [9].

The most common *NRAS*mt were *NRAS*-G12D, *NRAS*-Q61K, and *NRAS*-Q61R as seen in another study with a small number of patients [12]. Clinical characteristics, treatment, and OS among *NRAS*mt were similar, except for *NRAS*-Q61K being slightly older. One study has reported clinical characteristics for different *NRAS*mt, but the numbers were too small (*n* = 1–4 among different mutations) for drawing any conclusions [5], whereas other studies have only studied all *NRAS*mt as one group [4, 15, 27, 28].

The strengths of this study are that it is composed of two large population-based materials and one real-world material, which is, thus, reasonably representative of the background population. Eighty percent of the patients were molecularly characterised, making the results robust. The patient numbers were also sufficient for analysing the effect of most subgroups of *KRAS*mt and *NRAS*mt mutations. All three cohorts have very extensive and reliable data on clinical characteristics and treatments given, which makes it possible to analyse the effect of these factors on the outcome in great detail.

The extent of patient selection in patient materials is hard to evaluate, but a higher proportion of right-sided primary tumours, more females, older age, and more patients receiving BSC only usually indicate less selection. The intent in the Uppsala region and the PRCRC-study cohorts was to minimize selection by including as many as possible prospectively and identifying the rest of the patients retrospectively; most probably, all *in vivo* diagnosed mCRC patients were identified.

The limitations are that there will always be a selection in the molecular analyses, since not all cases will have sufficient material for analyses, especially in the case of elderly patients or in patients with rapidly progressing disease, which is the case for several patients in this study. If all patients had been analysed, it is possible that *KRAS*mt and *NRAS*mt frequencies would have been slightly less common, whereas *BRAF*-V600Emt would have been even more common. A further weakness in the molecular testing is that it was done in clinical routine in two of the cohorts; however, according to ESMO-guidelines [37, 38]. Thus, some patients in the Uppsala region and the RAXO-study were not tested for rarer *KRAS* mutations (codons 117 and 146), *NRAS* and *BRAF* mutations, which slightly overestimates the proportions of *KRAS*mt in codons 12 and 13 among all *KRAS*mt, but not among all patients. Ideally, a single comprehensive testing method covering all genes would be scientifically preferable; however, the current approach reflects the clinical reality better. Limitations of the fitness for intense therapy analysis are that it was done retrospectively and did not include, for example, comorbidity, which would have been a relevant parameter.

## Conclusion

In these population-based and real-world mCRC cohorts, which reflect the background population well and better than clinical trials/hospital-based patient series, *KRAS*mt were found in 49% and *NRAS*mt in 4%. No clinically relevant differences in characteristics, treatment, or outcome could be seen between different *KRAS*mt and *NRAS*mt, indicating similar clinical behaviour and prognosis. With many drugs under development for specific RASmt and pan-RAS inhibitors an improvement in outcome will hopefully be seen for this large group of patients where many could derive benefit.

### Congresses

This work has been presented in part at the European Society for Medical Oncology World Congress on Gastrointestinal Cancer 2023, taking place 28 June to 1 July 2023, in Barcelona, Spain.

## Supplementary information

Supplementary material

## Data Availability

The data collected for this study can be made available to others in de-identified form after all primary and secondary endpoints have been published, in the presence of a data transfer agreement, and if the purpose of use complies with Nordic legislation. Requests for data sharing can be made to the corresponding author, including a proposal that must be approved by the steering committee.
