# Supplementary material for: KRAS and NRAS mutations in Nordic population-based and real-world metastatic colorectal cancer cohorts
Source: BJC Rep. 2025 Oct 21;3:72. doi: 10.1038/s44276-025-00188-5 (PMC12540680; doi:10.1038/s44276-025-00188-5)
Supplement: Supplementary file 1 — Supplementary material [file 44276_2025_188_MOESM1_ESM.pdf]

Table S1. *KRAS* mutations in the present and selected other metastatic colorectal cancer cohorts

	Present study				Modest, Ann Oncol 2016				Ottaiano, Cancers 2020				Jones, Br J Cancer 2017				Neumann, Pathol Res Pract 2009			
	N	% of *KRAS*mt	% of all	mOS	N	% of *KRAS*mt	% of all	mOS	N	% of *KRAS*mt	% of all	mOS	N	% of *KRAS*mt	% of all	mOS	N	% of *KRAS*mt	% of all	mOS
Total	2118	-	100 %	21.1	1239	-	100 %	-	446	-	100 %	-	392	-	100 %	31.3	1018	-	100 %	-
***KRAS*mt**	1033	100 %	49 %	21.4	462	100 %	37 %	21.0	192	100 %	43 %	-	168	100 %	43 %	25.8	400	100 %	39 %	-
G12D	297	29 %	14 %	20.1	152	33 %	12 %	25.2	57	30 %	13 %	11.6	49	29 %	13 %	-	144	36 %	14 %	-
G12V	231	22 %	11 %	23.8	92	20 %	7 %	23.5	38	20 %	9 %	17.3	41	24 %	10 %	24.9	87	22 %	9 %	-
G13D	179	17 %	8 %	20.5	71	15 %	6 %	17.6	35	18 %	8 %	27.0	26	15 %	7 %	-	75	19 %	7 %	-
G12C	60	6 %	3 %	23.1	28	6 %	2 %	16.8	13	7 %	3 %	7.3	15	9 %	4 %	24.9	32	8 %	3 %	-
G12A	54	5 %	3 %	20.6	22	5 %	2 %	24.0	14	7 %	3 %	16.3	15	9 %	4 %	-	24	6 %	2 %	-
G12S	52	5 %	2 %	14.8	34	7 %	3 %	19.1	12	6 %	3 %	5.0	16	10 %	4 %	-	26	7 %	3 %	-
A146T	47	5 %	2 %	21.6	18	4 %	1 %	-	8	4 %	2 %	19.3								
Q61H	26	3 %	1 %	17.7	18	4 %	1 %	-					4	2 %	1 %	-				
Q61L	15	1 %	1 %	-																
G12R	14	1 %	1 %	-	2	0 %	0 %	-									5	1 %	0 %	-
A146V	10	1 %	0.5 %	-	8	2 %	1 %	-	3	2 %	1 %	2.0								
G13C	9	1 %	0.4 %	-	2	0 %	0 %	-	3	2 %	1 %	29.8	2	1 %	1 %	-				
K117N	7	1 %	0.3 %	-					3	2 %	1 %	4.6								
Q61R	6	1 %	0.3 %	-																
A146P	5	0 %	0.2 %	-																
A59T	4	0 %	0.2 %	-																
Q61K	4	0 %	0.2 %	-																
G13R	3	0 %	0.1 %	-					2	1 %	0 %	-								
G13E	2	0 %	0.1 %	-																
G13A	1	0 %	0.0 %	-																
G13F	1	0 %	0.0 %	-																
G13V	1	0 %	0.0 %	-																
A59G	1	0 %	0.0 %	-	2	0 %	0 %	-												
***NRAS*mt**	82	100 %	4 %	26.3	38	100 %	3 %	21.3	14	100 %	3 %	-								
G12D	18	22 %	1 %	22.0																
Q61K	17	21 %	1 %	20.2																
Q61R	17	21 %	1 %	26.9					1	7 %	0 %	-								
G13R	8	10 %	0.4 %	-																
Q61L	5	6 %	0.2 %	-					1	7 %	0 %	-								
G13D	4	5 %	0.2 %	-																
Q61H	4	5 %	0.2 %	-					4	29 %	1 %	9.0								
G12C	3	4 %	0.1 %	-					4	29 %	1 %	4.8								
G12V	3	4 %	0.1 %	-																
G12R	2	2 %	0.1 %	-					2	14 %	0 %	-								
G61L	1	1 %	0.0 %	-																
**RAS&*BRAF*wt**	696	-	33 %	28.3	664	-	54 %	26.9												
***BRAF*-V600Emt**	307	-	14 %	9.2	74	-	6 %	11.7												

	Present study				Giampieri, Front Oncol 2021				Modest, Oncology 2012				Patelli, ESMO Open 2021				Chida, Oncologist 2021			
	N	% of *KRAS*mt	% of all	mOS	N	% of *KRAS*mt	% of all	mOS	N	% of *KRAS*mt	% of all	mOS	N	% of *KRAS*mt	% of all	mOS	N	% of *KRAS*mt	% of all	mOS
Total	2118	-	100 %	21.1	-	-	-	-	-	-	-	-	15633	-	100 %	-	1717	-	100 %	-
***KRAS*mt**	1033	100 %	49 %	21.4	120	100 %	-	-	119	100 %	-	-	5079	100 %	32 %	-	702	100 %	41 %	26.8
G12D	297	29 %	14 %	20.1	48	40 %	-	25.0	44	37 %	-	23.3	1705	34 %	11 %	-	261	37 %	15 %	-
G12V	231	22 %	11 %	23.8	32	27 %	-	21.2	36	30 %	-	18.4	1059	21 %	7 %	-	151	22 %	9 %	-
G13D	179	17 %	8 %	20.5	6	5 %	-	-					997	20 %	6 %	-	160	23 %	9 %	-
G12C	60	6 %	3 %	23.1	15	13 %	-	37.3	13	11 %	-	14.3	414	8 %	3 %	-	45	6 %	3 %	21.1
G12A	54	5 %	3 %	20.6	5	4 %	-	-	14	12 %	-	17.9	270	5 %	2 %	-	31	4 %	2 %	-
G12S	52	5 %	2 %	14.8	3	3 %	-	-	8	7 %	-	15.2	286	6 %	2 %	-	36	5 %	2 %	-
A146T	47	5 %	2 %	21.6	1	1 %	-	-												
Q61H	26	3 %	1 %	17.7																
Q61L	15	1 %	1 %		1	1 %	-	-												
G12R	14	1 %	1 %		1	1 %	-	-	4	3 %	-	15.5								
A146V	10	1 %	0.5 %		1	1 %	-	-												
G13C	9	1 %	0.4 %																	
K117N	7	1 %	0.3 %																	
Q61R	6	1 %	0.3 %																	
A146P	5	0 %	0.2 %		1	1 %	-	-												
A59T	4	0 %	0.2 %																	
Q61K	4	0 %	0.2 %																	
G13R	3	0 %	0.1 %																	
G13E	2	0 %	0.1 %																	
G13A	1	0 %	0.0 %																	
G13F	1	0 %	0.0 %																	
G13V	1	0 %	0.0 %																	
A59G	1	0 %	0.0 %																	
***NRAS*mt**	82	100 %	4 %	26.3									24/340	-	8 %	-				
G12D	18	22 %	1 %	22.0																
Q61K	17	21 %	1 %	20.2																
Q61R	17	21 %	1 %	26.9																
G13R	8	10 %	0.4 %																	
Q61L	5	6 %	0.2 %																	
G13D	4	5 %	0.2 %																	
Q61H	4	5 %	0.2 %																	
G12C	3	4 %	0.1 %																	
G12V	3	4 %	0.1 %																	
G12R	2	2 %	0.1 %																	
G61L	1	1 %	0.0 %																	
**RAS&*BRAF*wt**	696	-	33 %	28.3																
***BRAF*-V600Emt**	307	-	14 %	9.2													43	-	-	12.2

	Present study				He, Onco Targets Ther 2020				Zocche, Front Genet 2015				Lavacchi, Front Oncol 2022				Alawawdeh, Targeted Oncology 2022			
	N	% of *KRAS*mt	% of all	mOS	N	% of *KRAS*mt	% of all	mOS	N	% of *KRAS*mt	% of all	mOS	N	% of *KRAS*mt	% of all	mOS	N	% of *KRAS*mt	% of all	mOS
Total	2118	-	100 %	21.1	200	-	100 %	-	148	-	100 %	27	-	-	-	-	2305	-	100 %	
***KRAS*mt**	1033	100 %	49 %	21.4	82	100 %	41 %	-	48	100 %	32 %	24	183	100 %	-	21.5	772	100 %	33 %	
G12D	297	29 %	14 %	20.1	23	28 %	12 %	-	8	17 %	5 %	30	51	28 %	-	-	131	17 %	6 %	21
G12V	231	22 %	11 %	23.8	18	22 %	9 %	-	13	27 %	9 %	25	40	22 %	-	-	161	21 %	7 %	24
G13D	179	17 %	8 %	20.5	11	13 %	6 %	-	6	13 %	4 %	18	40	22 %	-	-	79	10 %	3 %	24
G12C	60	6 %	3 %	23.1	2	2 %	1 %	-					13	7 %	-	52.9	60	8 %	3 %	24
G12A	54	5 %	3 %	20.6	3	4 %	2 %	-									89	12 %	4 %	25
G12S	52	5 %	2 %	14.8	5	6 %	3 %	-	4	8 %	3 %	11					34	4 %	1 %	30
A146T	47	5 %	2 %	21.6	4	5 %	2 %	-												
Q61H	26	3 %	1 %	17.7	1	1 %	1 %	-					2	1 %	-	-				
Q61L	15	1 %	1 %		2	2 %	1 %	-												
G12R	14	1 %	1 %		1	1 %	1 %	-												
A146V	10	1 %	0.5 %																	
G13C	9	1 %	0.4 %		1	1 %	1 %	-					1	1 %	-	-				
K117N	7	1 %	0.3 %		2	2 %	1 %	-					2	1 %	-	-				
Q61R	6	1 %	0.3 %										2	1 %	-	-				
A146P	5	0 %	0.2 %																	
A59T	4	0 %	0.2 %		1	1 %	1 %	-					2	1 %	-	-				
Q61K	4	0 %	0.2 %																	
G13R	3	0 %	0.1 %																	
G13E	2	0 %	0.1 %										1	1 %	-	-				
G13A	1	0 %	0.0 %		2	2 %	1 %	-									30	4 %	1 %	22
G13F	1	0 %	0.0 %																	
G13V	1	0 %	0.0 %										1	1 %	-	-				
A59G	1	0 %	0.0 %																	
***NRAS*mt**	82	100 %	4 %	26.3	8	100 %	4 %	-												
G12D	18	22 %	1 %	22.0	3	38 %	2 %	-												
Q61K	17	21 %	1 %	20.2	1	13 %	1 %	-												
Q61R	17	21 %	1 %	26.9	3	38 %	2 %	-												
G13R	8	10 %	0.4 %																	
Q61L	5	6 %	0.2 %																	
G13D	4	5 %	0.2 %																	
Q61H	4	5 %	0.2 %																	
G12C	3	4 %	0.1 %																	
G12V	3	4 %	0.1 %																	
G12R	2	2 %	0.1 %																	
G61L	1	1 %	0.0 %																	
**RAS&*BRAF*wt**	696	-	33 %	28.3	88	-	44 %	-												
***BRAF*-V600Emt**	307	-	14 %	9.2	21	-	11 %	-												

Table S2. *KRAS* mutations in the present cohort and a mix of metastatic and non-metastatic other colorectal cancer cohorts

	Present study				Vaughn, Genes Chromosomes Cancer 2011				Araujo, BMC Cancer 2021				Li, Int J Clin Exp Pathol 2019				Imamura, Clin Cancer Res 2012				Koulouridi, Cancers 2022			
	N	% of *KRAS*mt	% of all	mOS	N	% of *KRAS*mt	% of all	mOS	N	% of *KRAS*mt	% of all	mOS	N	% of *KRAS*mt	% of all	mOS	N	% of *KRAS*mt	% of all	mOS	N	% of *KRAS*mt	% of all	mOS
Total	2118	-	100 %	21.1	2121	-	100 %	-	4897	-	100 %	-	1164	-	100 %	-	1261	-	100 %	-	-	-	-	-
***KRAS*mt**	1033	100 %	49 %	21.4	900	100 %	42 %	-	2354	100 %	48 %	-	490	100 %	42 %	-	451	100 %	36 %	-	539	100 %	-	-
G12D	297	29 %	14 %	20.1	283	31 %	13 %	-	731	31 %	15 %	-	169	34 %	15 %	-	161	36 %	13 %	-	190	35 %	-	-
G12V	231	22 %	11 %	23.8	228	25 %	11 %	-	522	22 %	11 %	-	109	22 %	9 %	-	95	21 %	8 %	-	121	22 %	-	-
G13D	179	17 %	8 %	20.5	191	21 %	9 %	-					108	22 %	9 %	-	110	24 %	9 %	-	96	18 %	-	-
G12C	60	6 %	3 %	23.1	74	8 %	3 %	-	167	7 %	3 %	-	29	6 %	2 %	-	44	10 %	3 %	-	28	5 %	-	-
G12A	54	5 %	3 %	20.6	42	5 %	2 %	-					20	4 %	2 %	-					21	4 %	-	-
G12S	52	5 %	2 %	14.8	54	6 %	3 %	-					25	5 %	2 %	-	12	3 %	1 %	-	27	5 %	-	-
A146T	47	5 %	2 %	21.6																	15	3 %	-	-
Q61H	26	3 %	1 %	17.7	9	1 %	0 %	-													5	1 %	-	-
Q61L	15	1 %	1 %		3	0 %	0 %	-													2	0 %	-	-
G12R	14	1 %	1 %										4	1 %	0 %	-	8	2 %	1 %	-	3	1 %	-	-
A146V	10	1 %	0.5 %																		6	1 %	-	-
G13C	9	1 %	0.4 %		5	1 %	0 %	-									3	1 %	0 %	-				
K117N	7	1 %	0.3 %																		2	0 %	-	-
Q61R	6	1 %	0.3 %																		2	0 %		
A146P	5	0 %	0.2 %																					
A59T	4	0 %	0.2 %																		2	0 %	-	-
Q61K	4	0 %	0.2 %																					
G13R	3	0 %	0.1 %		1	0 %	0 %	-													3	1 %	-	-
G13E	2	0 %	0.1 %		2	0 %	0 %	-																
G13A	1	0 %	0.0 %																					
G13F	1	0 %	0.0 %																					
G13V	1	0 %	0.0 %		1	0 %	0 %	-									2	0 %	0 %	-				
A59G	1	0 %	0.0 %																					
***NRAS*mt**	82	100 %	4 %	26.3																	39	100 %	-	-
G12D	18	22 %	1 %	22.0																	8	21 %	-	-
Q61K	17	21 %	1 %	20.2																	7	18 %	-	-
Q61R	17	21 %	1 %	26.9																	9	23 %	-	-
G13R	8	10 %	0.4 %																		2	5 %	-	-
Q61L	5	6 %	0.2 %																		2	5 %	-	-
G13D	4	5 %	0.2 %																					
Q61H	4	5 %	0.2 %																		1	3 %	-	-
G12C	3	4 %	0.1 %																					
G12V	3	4 %	0.1 %																					
G12R	2	2 %	0.1 %																					
G61L	1	1 %	0.0 %																					
**RAS&*BRAF*wt**	696	-	33 %	28.3																				
***BRAF*-V600Emt**	307	-	14 %	9.2																				

Table S3. Clinical characteristics among all patients in the cohorts

		Total		RAXO		Uppsala		PRCRC		p-value
		2649	100 %	1086	41 %	765	29 %	798	30 %	
Age groups	≤70 years	1404	53 %	715	66 %	313	41 %	376	47 %	<0.001
	>70 years	1245	47 %	371	34 %	452	59 %	422	53 %	
Sex	Male	1479	56 %	656	60 %	405	53 %	418	52 %	<0.001
	Female	1170	44 %	430	40 %	360	47 %	380	48 %	
Primary tumour location	Right colon	879	34 %	310	29 %	291	38 %	278	36 %	<0.001
	Left colon	855	33 %	396	37 %	207	27 %	252	33 %	
	Rectum	881	34 %	374	35 %	262	34 %	245	32 %	
	Multiple/unknown	34	-	6	-	5	-	23	-	-
Primary resection	No	812	31 %	250	23 %	339	44 %	223	28 %	<0.001
	Yes	1837	69 %	836	77 %	426	56 %	575	72 %	
Presentation of metastases	Synchronous	1680	63 %	735	68 %	512	67 %	433	54 %	<0.001
	Metachronous	969	37 %	351	32 %	253	33 %	365	46 %	
Number of metastatic sites	1	1231	46 %	586	54 %	367	48 %	278	35 %	<0.001
	2	918	35 %	319	29 %	273	36 %	326	41 %	
	3-6	500	19 %	181	17 %	125	16 %	194	24 %	
ECOG PS	0	797	30 %	295	27 %	252	33 %	250	31 %	<0.001
	1	1075	41 %	600	55 %	243	32 %	232	29 %	
	2-4	773	29 %	191	18 %	269	35 %	313	39 %	
	Not available	4	-	0	-	-	-	3	-	-
Molecular testing	Yes	2115	80 %	927	85 %	704	92 %	484	61 %	<0.001
	No	534	20 %	159	15 %	61	8 %	314	39 %	
Type of treatment	Metastasectomy	659	25 %	397	37 %	213	28 %	49	6 %	<0.001
	Systemic therapy only	1411	53 %	666	61 %	334	44 %	411	52 %	
	Best supportive care	578	22 %	23	2 %	217	28 %	338	42 %	
	Not available	-	-	-	-	1	-	-	-	-
Type of systemic therapy	Biologic therapy	1277	63 %	869	82 %	266	52 %	142	31 %	<0.001
	No biologic therapy	746	37 %	189	18 %	242	48 %	315	69 %	
Overall survival	Median, months (95% CI)	18.7 (17.6-19.9)		30.0 (28.0-32.1)		15.1 (13.1-17.1)		9.8 (8.5-11.0)		<0.001
	1-year rate	63 %		82 %		57 %		43 %		-
	3-year rate	28 %		42 %		25 %		11 %		-
	Systemic therapy only	17.5 (16.5-18.6)		20.8 (19.3-22-3)		13.8 (12.2-15.5)		15.0 (13.8-16.1)		<0.001
	Cytotoxics only	11.1 (10.1-12.1)		10.8 (7.7-13.8)		10.2 (8.7-11.7)		11.9 (10.4-13.3)		0.064
	Bevacizumab/EGFR-inhibitor	22.6 (21.1-24.1)		23.2 (21.2-25.2)		20.5 (17.1-23.8)		23.9 (20.0-27.8)		0.218
	Metastasectomy and/or LAT	68.3 (61.0-75.6)		71.5 (62.8-80.2)		63.2 (53.8-72.5)		60.5 (51.1-69.9)		0.516
	Best supportive care	3.4 (3.0-3.8)		2.9 (2.6-3.1)		4.3 (3.4-5.2)		3.2 (2.7-3.7)		0.029

CI=confidence interval, ECOG PS=Eastern Cooperative Oncology Group performance status, LAT=local ablative therapy

Table S4. Clinical characteristics of patients with molecularly adequately tested patients in the cohorts

		Total		RAXO		Uppsala		PRCRC		p-value
		2118	100 %	927	44 %	705	33 %	486	23 %	
Age groups	≤70 years	1168	55 %	617	67 %	301	43 %	250	51 %	<0.001
	>70 years	950	45 %	310	33 %	404	57 %	236	49 %	
Sex	Male	1180	56 %	562	61 %	374	53 %	244	50 %	<0.001
	Female	938	44 %	365	39 %	331	47 %	242	50 %	
Primary tumour location	Right colon	712	34 %	267	29 %	261	37 %	184	38 %	<0.001
	Left colon	695	33 %	331	36 %	194	28 %	170	36 %	
	Rectum	695	33 %	324	35 %	247	35 %	124	26 %	
	Multiple/unknown	16	-	5	-	3	-	8	-	-
Primary resection	No	548	26 %	216	23 %	291	41 %	41	8 %	<0.001
	Yes	1570	74 %	711	77 %	414	59 %	445	92 %	
Presentation of metastases	Synchronous	1306	62 %	630	68 %	459	65 %	217	45 %	<0.001
	Metachronous	812	38 %	297	32 %	246	35 %	269	55 %	
Number of metastatic sites	1	1034	49 %	492	53 %	336	48 %	206	42 %	<0.001
	2	709	33 %	272	29 %	251	36 %	186	38 %	
	3-5	375	18 %	163	18 %	118	17 %	94	19 %	
ECOG PS	0	685	32 %	259	28 %	249	35 %	177	36 %	<0.001
	1	880	42 %	510	55 %	221	31 %	149	31 %	
	2-4	552	26 %	158	17 %	235	33 %	159	33 %	
	Not available	1	-	-	-	-	-	1	-	-
Mutation groups	*KRAS* mt	1033	49 %	492	53 %	331	47 %	210	43 %	<0.001
	NRAS mt	82	4 %	37	4 %	27	4 %	18	4 %	
	RAS&*BRAF* wt	696	33 %	304	33 %	224	32 %	168	35 %	
	BRAF-V600E mt	307	14 %	94	10 %	123	17 %	90	19 %	
Type of treatment	Metastasectomy	586	28 %	341	37 %	209	30 %	36	7 %	<0.001
	Systemic therapy only	1168	55 %	569	61 %	322	46 %	277	57 %	
	Best supportive care	364	17 %	17	2 %	174	25 %	173	36 %	
Type of systemic therapy	Biologic therapy	1110	65 %	753	83 %	262	53 %	95	31 %	<0.001
	No biologic therapy	598	35 %	153	17 %	230	47 %	215	69 %	
Overall survival	Median, months (95% CI)	21.1 (19.9-22.4)		30.5 (28.0-32.91)		16.5 (14.2-18.9)		11.8 (10.1-13.5)		<0.001
	1-year rate	68 %		83 %		60 %		50 %		-
	3-year rate	31 %		43 %		27 %		14 %		-
	Systemic therapy only	18.7 (17.5-19.9)		21.4 (19.5-22.8)		14.4 (12.7-16.1)		16.1 (14.3-17.9)		<0.001
	Cytotoxics only	11.8 (10.5-13.1)		13.7 (11.1-16.4)		10.3 (8.8-11.7)		13.0 (10.7-15.3)		0.006
	Bevacizumab/EGFR-inhibitor	23.2 (21.5-24.8)		23.2 (21.0-25.4)		20.5 (17.2-23.8)		26.9 (22.2-31.5)		0.173
	Metastasectomy and/or LAT	67.7 (30.1-75.2)		71.1 (61.3-81.0)		61.4 (53.2-69.6)		64.0 (19.9-108.1)		0.418
	Best supportive care	4.0 (3.3-4.8)		2.9 (2.1-3.7)		4.5 (3.5-5.6)		4.0 (3.3-4.8)		0.234

CI=confidence interval, ECOG PS=Eastern Cooperative Oncology Group performance status, LAT=local ablative therapy

Table S5. Systemic therapy used according to mutation status

		Total		*KRAS*mt		*NRAS*mt		RAS&*BRAF*wt		*BRAF*-V600Emt		p-value
		1708	100 %	843	49 %	71	4 %	577	34 %	217	13 %	
Number of lines	1	687	40 %	322	38 %	29	41 %	236	41 %	100	46 %	<0.001
	2	477	28 %	246	29 %	17	24 %	134	23 %	80	37 %	
	≥3	544	32 %	275	33 %	25	35 %	207	36 %	37	17 %	
First line chemotherapy	fluoropyrimidine	1674	98 %	830	98 %	70	99 %	563	98 %	211	97 %	0.528
	oxaliplatin	993	58 %	510	60 %	42	59 %	316	55 %	125	58 %	0.197
	irinotecan	446	26 %	187	22 %	18	25 %	181	31 %	60	28 %	0.002
	bevacizumab	688	40 %	381	45 %	36	51 %	187	32 %	84	39 %	<0.001
	EGFR-inhibitor	175	10 %	11	1 %	4	6 %	149	26 %	11	5 %	<0.001
Best response in first line	PR/CR/NED	871	56 %	409	53 %	36	60 %	353	66 %	73	38 %	<0.001
	SD	468	30 %	269	35 %	14	23 %	125	23 %	60	32 %	
	PD	223	14 %	96	12 %	10	17 %	60	11 %	57	30 %	
	Missing	146	-	69	-	11	-	39	-	27	-	-
Chemotherapy all lines	fluoropyrimidine	1686	99 %	835	99 %	70	99 %	567	98 %	214	99 %	0.640
	oxaliplatin	1290	76 %	656	78 %	57	80 %	421	73 %	156	72 %	0.076
	irinotecan	1175	69 %	579	69 %	48	68 %	409	71 %	139	64 %	0.321
	bevacizumab	916	54 %	496	59 %	42	59 %	280	49 %	98	45 %	<0.001
	EGFR-inhibitor	416	24 %	40	5 %	11	15 %	335	58 %	30	14 %	<0.001

CR=complete response, NED=no evidence of disease, PD=progressive disease, PR=partial response, SD=stable disease

Table S6. Fitness for intensive therapy in mutations groups, treatment groups according to guidelines, and among different *KRAS* mutants

		Total		Fit for intensive therapy*		Not eligible for intensive therapy					
						Total		Reasons for unfit			
								Age >75 years		ECOG PS 2-4	
		1190	100 %	565	47 %	625	53 %	432	36 %	394	33 %
**Mutation status**	*KRAS*mt	540	100 %	259	48 %	281	52 %	204	38 %	169	31 %
	*NRAS*mt	45	100 %	30	67 %	15	33 %	13	29 %	6	13 %
	RAS&*BRAF*wt	392	100 %	199	51 %	193	49 %	128	33 %	120	31 %
	BRAF-V600Emt	213	100 %	77	36 %	136	64 %	87	41 %	99	46 %
**Treatment groups**	Left-sided RAS&*BRAF*wt	297	100 %	158	53 %	139	47 %	89	30 %	89	30 %
	Right-sided RAS&*BRAF*wt	81	100 %	34	42 %	47	58 %	34	42 %	26	32 %
	*KRAS*mt	525	100 %	252	48 %	273	52 %	199	38 %	165	31 %
	*NRAS*mt	44	100 %	29	66 %	15	34 %	13	30 %	6	14 %
	BRAF-V600Emt	154	100 %	58	38 %	96	62 %	56	36 %	68	44 %
	dMMR	89	100 %	34	38 %	55	62 %	41	46 %	40	45 %
***KRAS*mt**	G12D	168	100 %	75	45 %	93	55 %	73	43 %	49	29 %
	G12V	113	100 %	54	48 %	59	52 %	44	39 %	32	28 %
	G12C	26	100 %	7	27 %	19	73 %	11	42 %	16	62 %
	G12A	30	100 %	18	60 %	12	40 %	9	30 %	9	30 %
	G12S	29	100 %	18	62 %	11	38 %	7	24 %	9	31 %
	G13D	91	100 %	43	47 %	48	53 %	33	36 %	28	31 %
	Q61H	13	100 %	6	46 %	7	54 %	5	38 %	4	31 %
	A146T	24	100 %	15	63 %	9	38 %	8	33 %	4	17 %

dMMR=deficient mismatch repair, ECOG PS=Eastern Cooperative Oncology Group performance status. Statistically significant differences in bold and italic. *Fit for doublet/triplet chemotherapy+biologic

Table S7. Systemic therapy used according to the most common *KRAS* mutations

		Total*		G12D		G12V		G12C		G12A		G12S		G13D		Q61H		A146T		p-value
		843	100 %	234	28 %	196	19 %	46	4 %	45	4 %	43	4 %	151	15 %	19	2 %	37	4 %	
Number of lines	1	322	38 %	75	32 %	80	41 %	18	39 %	16	36 %	21	49 %	68	45 %	8	42 %	9	24 %	0.019
	2	246	29 %	85	36 %	48	24 %	16	35 %	12	27 %	10	23 %	35	23 %	10	53 %	15	41 %	
	≥3	275	33 %	74	32 %	68	35 %	12	26 %	17	38 %	12	28 %	48	32 %	1	5 %	13	35 %	
First line chemotherapy	fluoropyrimidine	830	98 %	230	98 %	193	98 %	45	98 %	44	98 %	43	100 %	148	98 %	19	100 %	36	97 %	0.981
	oxaliplatin	510	60 %	139	59 %	115	59 %	27	59 %	26	58 %	31	72 %	89	59 %	11	58 %	21	57 %	0.875
	irinotecan	187	22 %	56	24 %	41	21 %	13	28 %	11	24 %	7	16 %	32	21 %	6	32 %	10	27 %	0.794
	bevacizumab	381	45 %	96	41 %	89	45 %	26	57 %	18	40 %	17	40 %	73	48 %	12	63 %	20	54 %	0.233
Best response in first line	PR/CR/NED	409	53 %	117	55 %	91	51 %	19	46 %	20	49 %	21	55 %	77	54 %	10	53 %	19	51 %	0.744
	SD	269	35 %	70	33 %	74	41 %	15	37 %	15	37 %	11	29 %	42	30 %	7	37 %	14	38 %	
	PD	96	12 %	26	12 %	15	8 %	7	17 %	6	15 %	6	16 %	23	16 %	2	11 %	4	11 %	
	Missing	69	-	21	-	16	-	5	-	4	-	5	-	9	-	-	-	-	-	-
Chemotherapy all lines	fluoropyrimidine	835	99 %	230	98 %	194	99 %	46	100 %	45	100 %	43	100 %	149	99 %	19	100 %	37	100 %	0.872
	oxaliplatin	656	78 %	183	78 %	146	74 %	36	78 %	35	78 %	35	81 %	111	74 %	15	79 %	32	86 %	0.738
	irinotecan	579	69 %	168	72 %	134	68 %	32	70 %	30	67 %	27	63 %	97	64 %	15	79 %	30	81 %	0.450
	bevacizumab	496	59 %	137	59 %	114	58 %	32	70 %	20	44 %	19	44 %	84	56 %	16	84 %	29	78 %	0.003

*71 with less common *KRAS* mutations not presented separately and not included in chi-square analyses, CR=complete response, NED=no evidence of disease, PD=progressive disease, PR=partial response, SD=stable disease

Table S8. Clinical characteristics according to the most common *NRAS* mutations

		Total		G12D		Q61K		Q61R		p-value
		82	100 %	18	22 %	17	21 %	17	21 %	
Median age (range)		66 (30-92)		58 (43-86)		75 (30-92)		67 (46-79)		0.006
Total		82	100 %	18	100 %	17	100 %	17	100 %	-
Age groups	≤70 years	51	62 %	14	78 %	3	18 %	12	71 %	<0.001
	>70 years	31	38 %	4	22 %	14	82 %	5	29 %	
Sex	Male	46	56 %	12	67 %	9	53 %	9	53 %	0.635
	Female	36	44 %	6	33 %	8	47 %	8	47 %	
Primary tumour location	Right colon	10	12 %	0	0 %	4	24 %	4	24 %	0.222
	Left colon	30	37 %	7	39 %	6	35 %	7	41 %	
	Rectum	42	51 %	11	61 %	7	41 %	6	35 %	
Primary resection	No	23	28 %	7	39 %	1	6 %	6	35 %	0.057
	Yes	59	72 %	11	61 %	16	94 %	11	65 %	
Tumour grade	Low	58	83 %	12	86 %	14	82 %	12	92 %	0.731
	High	12	17 %	2	14 %	3	18 %	1	8 %	
	Not available	12	-	4	-	-	-	4	-	-
Presentation of metastases	Synchronous	53	65 %	14	78 %	5	29 %	15	88 %	<0.001
	Metachronous	29	35 %	4	22 %	12	71 %	2	12 %	
Number of metastatic sites	1	41	50 %	6	33 %	9	53 %	8	47 %	0.077
	2	25	30 %	9	50 %	6	35 %	2	12 %	
	3-6	16	20 %	3	17 %	2	12 %	7	41 %	
Metastatic sites	Liver	61	74 %	15	83 %	10	59 %	14	82 %	0.171
	Lung	28	34 %	6	33 %	7	41 %	5	29 %	0.763
	Lymph nodes	22	27 %	7	39 %	3	18 %	4	24 %	0.341
	Peritoneum	15	18 %	5	28 %	2	12 %	5	29 %	0.400
	Bone	2	2 %	0	0 %	2	12 %	0	0 %	0.118
	Other	11	13 %	1	6 %	3	18 %	3	18 %	0.478
ECOG PS	0	34	41 %	4	22 %	6	35 %	7	41 %	0.682
	1	35	43 %	9	50 %	7	41 %	8	47 %	
	2-4	13	16 %	5	28 %	4	24 %	2	12 %	
Smoking status	No	24	55 %	3	50 %	4	36 %	6	67 %	0.403
	Yes	20	45 %	3	50 %	7	64 %	3	33 %	
	Not available	38	-	12	-	6	-	8	-	-
Mismatch repair status	pMMR	43	93 %	13	93 %	11	92 %	6	100 %	0.776
	dMMR	3	7 %	1	7 %	1	8 %	0	0 %	
	Not tested	36	-	4	-	5	-	11	-	-
Type of treatment	Metastasectomy	29	35 %	4	22 %	5	29 %	5	29 %	0.365
	Systemic therapy only	46	56 %	13	72 %	8	47 %	11	65 %	
	Best supportive care	7	9 %	1	6 %	4	24 %	1	6 %	

*30 with less common *NRAS* mutations not presented separately and not included in chi-square analyses, dMMR=deficient mismatch repair, ECOG PS=Eastern Cooperative Oncology Group performance status, pMMR=proficient mismatch repair

Table S9. Systemic therapy used according to the most common *NRAS* mutations

		Total*		G12D		Q61K		Q61R		p-value
		71	4 %	16	23 %	12	17 %	14	20 %	
Number of lines	1	29	41 %	6	38 %	5	42 %	6	43 %	0.798
	2	17	24 %	4	25 %	4	33 %	2	14 %	
	≥3	25	35 %	6	38 %	3	25 %	6	43 %	
First line chemotherapy	fluoropyrimidine	70	99 %	15	94 %	12	100 %	14	100 %	0.435
	oxaliplatin	42	59 %	11	69 %	6	50 %	8	57 %	0.592
	irinotecan	18	25 %	4	25 %	1	8 %	3	21 %	0.519
	bevacizumab	36	51 %	6	38 %	6	50 %	9	64 %	0.343
Best response in first line	PR/CR/NED	36	60 %	6	46 %	7	64 %	7	64 %	0.591
	SD	14	23 %	3	23 %	1	9 %	3	27 %	
	PD	10	17 %	4	31 %	3	27 %	1	9 %	
	Missing	11	-	3	-	1	-	3	-	-
Chemotherapy all lines	fluoropyrimidine	70	99 %	15	94 %	12	100 %	14	100 %	0.435
	oxaliplatin	57	80 %	14	88 %	9	75 %	11	79 %	0.680
	irinotecan	48	68 %	11	69 %	7	58 %	8	57 %	0.772
	bevacizumab	42	59 %	9	56 %	6	50 %	10	71 %	0.510

*29 with less common *NRAS* mutations not presented separately and not included in chi-square analyses, CR=complete response, NED=no evidence of disease, PD=progressive disease, PR=partial response, SD=stable disease

Figure S1A-B. Progression free survival for mutations groups (A) and the most common *KRAS* mutants (B)

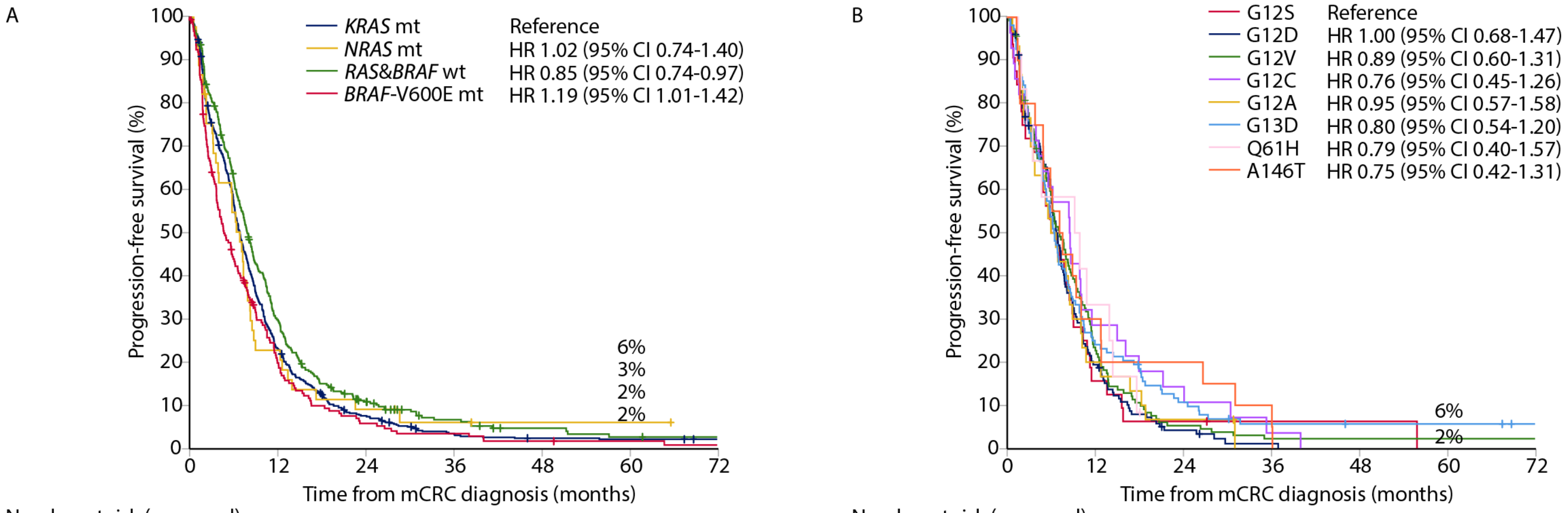

Number at risk (censored)

KRAS mt	564 (7)	129 (11)	40 (15)	16 (16)	10 (16)	9 (18)	7 (23)
NRAS mt	45 (1)	10 (1)	4 (2)	2 (3)	1 (3)	1 (4)	0 (4)
RAS&BRAF wt	361 (7)	105 (13)	33 (22)	14 (25)	7 (25)	4 (26)	3 (29)
BRAF-V600E mt	188 (8)	35 (8)	10 (8)	6 (8)	3 (9)	2 (9)	1 (10)

Number at risk (censored)

G12S	32 (0)	5 (0)	2 (1)	1 (1)	1 (1)	0 (1)	0 (1)
G12D	149 (4)	28 (6)	5 (7)	1 (7)	0 (7)	0 (7)	0 (7)
G12V	136 (3)	31 (3)	7 (3)	3 (3)	3 (3)	3 (3)	3 (6)
G12C	28 (0)	8 (0)	4 (0)	1 (0)	0 (0)	0 (0)	0 (0)
G12A	30 (0)	6 (0)	2 (1)	0 (1)	0 (1)	0 (1)	0 (1)
G13D	108 (0)	26 (1)	11 (2)	5 (3)	4 (3)	4 (5)	2 (6)
Q61H	12 (0)	4 (1)	0 (1)	0 (1)	0 (1)	0 (1)	0 (1)
A146T	20 (0)	6 (0)	4 (0)	2 (0)	0 (0)	0 (0)	0 (0)

Figure S2. Overall survival for the most common *NRAS* mutations

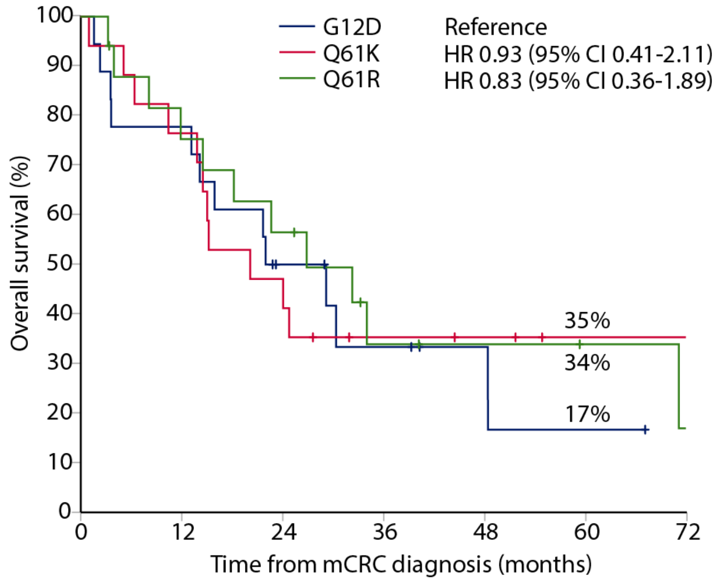

Number at risk (censored)

G12D	18 (0)	14 (2)	7 (3)	4 (5)	2 (5)	1 (6)	0 (6)
Q61K	17 (0)	13 (0)	8 (2)	4 (3)	3 (5)	1 (5)	1 (6)
Q61R	17 (1)	12 (1)	9 (1)	4 (4)	3 (5)	2 (5)	1 (6)
